# Low lymphocyte‐to‐monocyte ratio predicts poor outcome in high‐risk aggressive large B‐cell lymphoma

**DOI:** 10.1002/jha2.409

**Published:** 2022-06-23

**Authors:** Heli Vajavaara, Suvi‐Katri Leivonen, Judit Jørgensen, Harald Holte, Sirpa Leppä

**Affiliations:** ^1^ Research Program Unit Applied Tumor Genomics Faculty of Medicine University of Helsinki Helsinki Finland; ^2^ Department of Oncology Helsinki University Hospital Comprehensive Cancer Center Helsinki Finland; ^3^ iCAN Digital Precision Cancer Medicine Flagship Helsinki Finland; ^4^ Department of Hematology Aarhus University Hospital Aarhus Denmark; ^5^ Department of Oncology KG Jebsen Center for B‐Cell Malignancies Oslo University Hospital Oslo Norway

**Keywords:** diffuse large B‐cell lymphoma, lymphocyte count, monocytes, prognosis, prospective studies

## Abstract

Low lymphocyte‐to‐monocyte‐ratio (LMR) has been associated with unfavorable survival in patients with diffuse large B‐cell lymphoma (DLBCL). To date, however, the impact of LMR on survival has not been examined in a uniformly treated cohort of patients with high‐risk aggressive large B‐cell lymphoma. We collected peripheral blood absolute lymphocyte counts (ALCs) and absolute monocyte counts (AMC) prior to treatment and calculated LMR from 112 adult patients, who were less than 65 years of age, had age‐adjusted International Prognostic Index 2–3, or site‐specific risk factors for central nervous system (CNS) recurrence, and were treated in a Nordic Lymphoma Group LBC‐05 trial with dose‐dense immunochemotherapy and early systemic CNS prophylaxis (www.ClinicalTrials.gov, number NCT01325194). Median pretreatment ALC was 1.40 × 10^9^/l (range, 0.20–4.95), AMC 0.68 × 10^9^/l (range, 0.10–2.62), and LMR 2.08 (range, 0.10–12.00). ALC did not correlate with tumor‐infiltrating lymphocytes, AMC did not correlate with tumor‐associated macrophages, and neither ALC nor AMC correlated with survival. However, low LMR (<1.72) translated to unfavourable progression‐free survival (PFS) (5‐year PFS 70% vs. 92%, *p = *0.002) and overall survival (OS) (5‐year OS, 77% vs. 92%, *p = *0.020). In the patients with low LMR, relative risk of progression was 4.4‐fold (95% confidence interval [CI] 1.60–12.14, *p = *0.004), and relative risk of death was 3.3‐fold (95% CI 1.18–9.50, *p = *0.024) in comparison to the patients with high LMR. We conclude that low LMR is an adverse prognostic factor in uniformly treated young patients with high‐risk aggressive large B‐cell lymphoma.

## 1 INTRODUCTION

Combination of rituximab (R) with anthracycline‐based chemotherapy has improved the outcome of patients with diffuse large B‐cell lymphoma (DLBCL) [1, 2, 3]. Nevertheless, approximately one third of the patients have primary refractory disease or they relapse and ultimately die from lymphoma. International Prognostic Index (IPI), which is based on five clinical risk factors, has remained the main prognostic tool to stratify patients into low‐, intermediate‐, and high‐risk groups [4]. The risk factors in the IPI classification are age >60 years, stage >II, Eastern Cooperative Oncology Group (ECOG) performance status >1, lactate dehydrogenase (LDH) level > upper limit of normal, and >1 extranodal site. As the IPI was designed before rituximab era, other prognostic tools such as revised‐IPI [5] (IPI) and National Comprehensive Cancer Network‐IPI [6] (NCCN‐IPI) have been developed. However, they are based on the same clinical parameters as the original IPI, and none of the three indexes can accurately identify ultrahigh‐risk patients [7]. In addition, the outcome within the individual IPI risk groups can vary considerably, indicating that the biological features are not captured by the different IPIs.

Apart from the clinical risk factors, biological prognostic markers have also been studied. DLBCL not otherwise specified (NOS) can be divided into germinal center B‐cell like (GCB) and activated B‐cell like (ABC) molecular subtypes according to cell of origin. Of them, ABC‐like DLBCLs have worse prognosis than GCB‐DLBCLs, although the data are not fully consistent [7]. Double hit lymphomas with *BCL2* and *MYC* translocations, other double‐expressor lymphomas that overexpress Bcl2 and Myc proteins, and lymphomas containing genomic aberrations in the *TP53* gene also have inferior outcome [8, 9, 10, 11, 12]. Additionally, tumor infiltrating nonmalignant immune cells and gene expression signatures reflecting the activity of tumor microenvironment (TME) have an impact on the outcome [13, 14, 15, 16, 17]. Despite promising results, many of these biomarkers are difficult to implement to routine clinical practice, and there is a need for easily applicable and widely available prognostic tools.

The lymphocyte/monocyte ratio (LMR), calculated by the ratio of absolute lymphocyte count (ALC) to absolute monocyte count (AMC), is an inflammatory biomarker indicating the balance between the host immune system and TME. Low ALC, high AMC, and low LMR have been shown to translate to poor survival in patients with DLBCL [18, 19, 20, 21, 22, 23, 24, 25, 26, 27, 28, 29, 30, 31, 32, 33]. To our knowledge, however, the prognostic impact of LMR has been demonstrated only in heterogeneous patient populations treated mostly with cyclophosphamide, doxorubicin, vincristine, and prednisone (CHOP) or R‐CHOP. The main purpose of our study was to examine if LMR has prognostic impact on survival in a uniformly treated cohort of young patients with high‐risk aggressive large B‐cell lymphoma. Secondarily, we investigated whether the blood values correlate to the lymphocyte and macrophage content in the TME examined in a previous study on the same patient cohort [15].

## 2 MATERIALS AND METHODS

### 2.1 Patients and samples

The study population consisted of 112 patients, 18–64 years of age with high‐risk (age‐adjusted IPI [aaIPI] 2–3 or site‐specific risk factors for central nervous system [CNS] recurrence) primary DLBCL NOS or other aggressive large B‐cell lymphoma with available pretreatment ALC and AMC values. The patients were treated in a Nordic Lymphoma Group (NLG) phase II LBC‐05 trial with biweekly rituximab, cyclophosphamide, doxorubicin, vincristine, etoposide, and prednisone (R‐CHOEP) immunochemotherapy and early systemic CNS prophylaxis (high‐dose [HD] methotrexate and HD‐cytarabine) [34]. All patients signed informed consent before study participation. The trial was registered at www.ClinicalTrials.gov, number: NCT01325194. National Medical Agencies, Institutional Review Boards, and Ethics Committees in Finland, Norway, Denmark, and Sweden approved the protocol and sampling.

Lymphocyte and monocyte values were obtained from routine automated complete blood count determination from peripheral blood samples drawn prior to treatment initiation. The values were collected retrospectively from the patient files. LMR was calculated by dividing ALC by AMC.

Matched soluble CD163 levels measured from serum with Quantikine enzyme‐linked immunosorbent assay (R&D Systems, Minneapolis, MN, USA) were available from 98 cases [35], and multiplex immunohistochemistry data on T lymphocyte (CD3+ and CD4+) and macrophage (CD68+ and CD163+) counts [15, 36] from 37 and 39 tumor samples, respectively. *CD3*, *CD68*, and *CD163* gene expression levels measured using digital gene expression analysis with NanoString nCounter (NanoString Technologies, Seattle, WA, USA) were available from 56, 58, and 58 tumor samples, respectively [15].

### 2.2 Statistical analyses

Statistical analyses were performed with IBM SPSS Statistics v.25.0 (IBM, Armonk, NY, USA), and R 4.0.2. Mann–Whitney *U* test was used to evaluate the difference of LMR level according to categorized or continuous clinical parameters. Correlations were evaluated with Spearman rank analysis. Chi‐square test was used to evaluate the differences in the frequency of the prognostic factors. To categorize patients into low and high LMR groups, maximally selected rank statistics test with the R “maxstat” package was used [37] with survival outcomes categorized into progression or death versus no progression or death. The prognostic impact was estimated by Cox univariate and bivariate regression analysis (confidence interval [CI] 95%) using categorized variables. The difference in survival between the patient groups was estimated using the Kaplan–Meier method. The degree of survival significance was calculated using log‐rank test. Overall survival (OS) was defined as the time from the date of trial entry until death from any cause. Progression‐free survival (PFS) was defined as the time from the date of trial entry until relapse or death. Both OS and PFS were defined in months. *p*‐Values were adjusted for the errors due to multiple testing using the FDR method. *p*‐Values ≤0.05 were considered significant. All statistical tests were two‐tailed.

## 3 RESULTS

### 3.1 Clinical characteristics

NLG‐LBC‐05 trial included 139 previously untreated patients, 18–64 years of age, who had DLBCL or other aggressive large B‐cell lymphoma entity with aaIPI 2–3 or site‐specific risk factors for CNS recurrence [34]. Baseline characteristics of the study cohort (*n* = 112) are summarized in Table 1. The median age was 56 years (range 22–64 years). Majority of the patients were males (64%), had B‐symptoms (66%), elevated LDH level (91%), and advanced stage disease (93%). According to national pathology review, the most common histology was DLBCL NOS (*n* = 94; 84%); the other subtypes were primary mediastinal B‐cell lymphoma (*n* = 6; 5.4%), grade 3B follicular lymphoma (*n* = 5; 4.5%), T‐cell rich B‐cell lymphoma (*n* = 4; 3.6%), and intravascular B‐cell lymphoma (*n* = 1; 0.9%). Two cases (1.8%) were not reviewed. Double‐hit lymphomas were found in seven (11%) of the examined 63 samples.

**TABLE 1:** Baseline characteristics of all patients and according to lymphocyte‐to‐monocyte level

Characteristic	*n* (%)	Low LMR, *n* (%)	High LMR, *n* (%)	*p*‐Value
*Total*	112 (100)	47 (42)	65 (58)	
*Median age (range)*	56 (22–65)	54 (22–65)	57 (22–65)	0.836
*Age*				
60 years	77 (69)	35 (75)	42 (65)	0.267
60–65 years	35 (31)	12 (25)	23 (35)	
*Gender*				
Male	72 (64)	36 (77)	36 (55)	0.021
Female	40 (36)	11 (23)	29 (45)	
*ECOG PS*				
0–1	77 (69)	29 (62)	48 (74)	0.171
2–4	35 (31)	18 (38)	17 (26)	
*Stage*				
1–2	8 (7)	2 (4)	6 (9)	0.313
3–4	104 (93)	45 (96)	59 (91)	
*aaIPI score*				
0–1	8 (7)	2 (4)	6 (9)	0.278
2	67 (60)	26 (55)	41 (63)	
3	37 (33)	19 (41)	18 (28)	
*Entity*				
DLBCL NOS				
GCB	48 (43)	18 (38)	30 (46)	0.293*
non‐GCB	39 (35)	19 (40)	20 (31)	
ND	7 (6)	3 (6)	4 (6)	
Other/missing	18 (16)	7 (14)	11 (17)	

*Note*: *p*‐Values between low and high LMR groups.

Abbreviations: aaIPI, age‐adjusted International Prognostic Index; ECOG PS, Eastern Cooperative Oncology Group performance status; DLBCL, diffuse large B‐cell lymphoma; GCB, germinal center B‐cell like; LMR, lymphocyte‐to‐monocyte‐ratio; ND, not determined; non‐GCB, non‐germinal center B‐cell like; NOS, not otherwise specified.

* Comparison between GCB and non‐CGB.

During the median follow‐up time of 61 months (range 27–85 months), 16 (14%) patients died, 11 (10%) due to lymphoma, and 17 (15%) experienced progression, translating to 86% 5‐year OS and 83% 5‐year PFS rates, respectively. There were no major differences in the baseline characteristics and survival between the patients originally included in the NLG‐LBC‐05 trial and the patients available for this study (data not shown), indicating that the patients were representative of the entire clinical trial cohort [34].

### 3.2 ALC, AMC, and LMR levels and their correlation with clinical parameters and gene and protein expression

**FIGURE 1:**
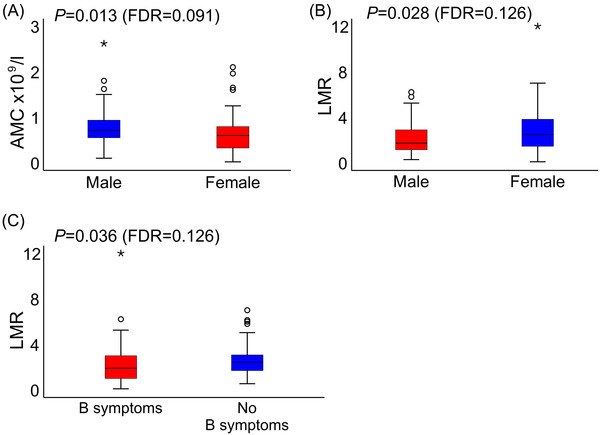
Association of absolute monocyte count (AMC) and lymphocyte‐to‐monocyte‐ratio (LMR) level with clinical factors. (A) Association of AMC with gender. Association of LMR with (B) gender and (C) B‐symptoms. FDR, false discovery rate

Median pretreatment ALC was 1.40 × 10^9^/l (range 0.20–4.95 × 10^9^/l), and median pretreatment AMC was 0.68 × 10^9^/l (range 0.10–2.62 × 10^9^/l), leading to a median LMR of 2.08 (range 0.10–12.00). High AMC associated with male gender (*p = *0.013; Figure 1A), while no correlation was observed between the AMC and B‐symptoms (*p *= 0.186), age (*p *= 0.620), stage (*p *= 0.699), LDH level (*p *= 0.117), ECOG performance status (*p *= 0.636), or molecular subtype (*p *= 0.313). ALC did not associate with gender (*p *= 0.633), B‐symptoms (*p *= 0.163), age (*p *= 0.774), stage (*p *= 0.218), LDH level (*p *= 0.470), ECOG performance status (*p *= 0.237), or molecular subtype (*p *= 0.442). Low LMR associated with male gender (*p = *0.028) and B‐symptoms (*p = *0.036) (Figure 1B,C), whereas no correlation was seen between the LMR and age (*p *= 0.247), stage (*p *= 0.182), LDH level (*p *= 0.561), ECOG performance status (*p *= 0.055), or molecular subtype (*p *= 0.224).

We observed a trend toward inverse correlation between the ALC and the proportion of tumor‐infiltrating T cells (*ρ* = −0.319, *p = *0.054, *n* = 37), while no correlation between the ALC and *CD3* gene expression levels in the tumor tissue was observed (*p *= 0.091). Likewise, inverse correlation was found between the ALC and CD4+ tumor‐infiltrating T cells (*ρ* = −0.346, *p = *0.036, *n* = 37). In contrast, AMC did not correlate with the proportion of tumor‐associated macrophages (TAMs) (*p *= 0.359). Neither did we observe any correlation between the AMC and *CD68* or *CD163* gene expression levels in the tumor tissue (*p *= 0.678 and *p *= 0.565, respectively) nor between the AMC and soluble CD163 levels in the serum (*p *= 0.998).

### 3.3 Association of LMR with survival

**FIGURE 2:**
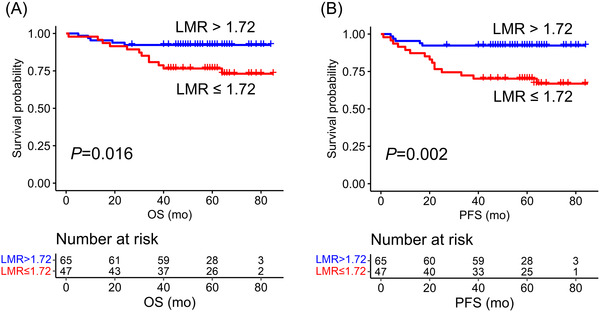
Survival association of lymphocyte‐to‐monocyte‐ratio (LMR). (A) Overall survival (OS) and (B) progression free survival (PFS) according to LMR level

According to maximally selected rank statistics, LMR cutoff level of 1.72 discriminated the patients into two subgroups (low LMR and high LMR) with different outcomes. The patients with low LMR (*n* = 47; 42%) had a poor outcome in comparison to the patients with high LMR (*n* = 65; 58%) (5‐year OS 77% vs. 92%, *p = *0.016; 5‐year PFS 70% vs. 92%, *p = *0.002; Figure 2A,B). Relative risk of death was 3.3‐fold (95% CI 1.18–9.50, *p = *0.024) and risk of progression 4.4‐fold (95% CI 1.60–12.14, *p = *0.004) in the low LMR group compared to the high LMR group.

There were more males in the low LMR group than in the high LMR group, whereas no differences in age, performance score, stage, aaIPI, or molecular subtype were observed between the LMR low and high subgroups (Table 1). When the LMR was adjusted for gender, the prognostic impact of LMR on survival was sustained (OS hazard ratio [HR] = 3.3, 95% CI 1.13–9.57, *p = *0.029; PFS, HR = 4.19, 95% CI 1.49–11.76, *p *= 0.007).

In bivariate analysis with aaIPI, LMR remained as a prognostic factor for OS (HR 3.11, 95% CI 1.08–8.92, *p = *0.035) and PFS (HR 4.45, 95% CI 1.49–11.53, *p = *0.006) (Table 2).

**TABLE 2:** Bivariate Cox regression analysis

	OS			PFS		
Factor	HR	95% CI	*p*‐Value	HR	95% CI	*p*‐value
LMR <1.72	3.11	1.08–8.92	**0.035**	4.45	1.49–11.53	**0.006**
aaIPI	1.49	0.66–3.34	0.340	1.37	0.66–2.85	0.397

Abbreviations: aaIPI, age‐adjusted International Prognostic Index; CI, confidence interval; HR, hazard ratio; LMR, lymphocyte‐to‐monocyte‐ratio; OS, overall survival; PFS, progression‐free survival.

ALC and AMC did not translate to survival (data not shown). Nevertheless, the ALC was more often reduced in the low LMR group than in the high LMR group (68% vs. 26%, respectively; *p* < 0.001), and the AMC was more often elevated (55% vs. 15%, respectively; *p* < 0.001).

## 4 DISCUSSION

Despite the previously identified tumor‐related biological prognostic factors, such as cell of origin [16, 38], *TP53* aberrations [10, 11], *BCL2* and *MYC* translocations and coexpression phenotype [8, 9, 10], or tumor infiltrating CD4+ lymphocytes [14, 15], age and IPI have remained the main tools in clinical practice to stratify the patients with DLBCL into low‐ and high‐risk groups and different therapies. Many of the biological prognostic factors, for example gene expression signatures, are not part of the routine diagnostic and stratification procedures.

Of the host‐derived factors, ALC and AMC are routinely measured pre‐therapeutically, and LMR can be calculated from these values by a simple mathematical division. Thus, determination of LMR does not cause any extra costs and is simply implemented. Low LMR has previously been associated with unfavorable survival in patients with DLBCL. Since the prognostic impact of LMR has to our knowledge neither been studied specifically in a high‐risk population, nor combined with comprehensive molecular data from the tumor tissue, we examined pre‐therapeutic ALC, AMC, and LMR in a cohort of patients with high‐risk aggressive B‐cell lymphoma, who were treated uniformly in a Nordic phase II trial. Although the number of relapses was low in our cohort, we found that LMR could separate the clinically high‐risk patients into two groups, the one having excellent and the other adverse outcome. Low LMR translated to poor OS and PFS, whereas low ALC or elevated AMC showed no correlation with survival.

Consistent with previous data [21], we also observed that high AMC and thus low LMR were more common in males than females. We speculate that the difference in the AMC between the sexes results from the difference of monocyte development in the bone marrow or monocyte recruitment to the tissues. A finding supporting this hypothesis is that upon stimulation with the gram‐negative stimulus lipopolysaccharide, monocyte concentrations have been found to be high in males compared to females [39]. Unlike in previous studies [21, 27], we did not, however, observe any correlation between low LMR and other clinical risk factors including IPI and advanced stage, presumably due to homogeneous patient material with small number of patients with low aaIPI (7%) and low stage (7%).

In our cohort, ALC and AMC did not as individual factors associate with outcome, although ALC was more often reduced, and AMC elevated in the low LMR group than in the high LMR group. While the result is opposite to some previous studies including patients from all risk groups [19, 22, 23, 24, 28, 40], similar findings have also been described [26, 41, 42]. Presumably due to a high‐risk patient population, the median AMC (0.68) was higher in our cohort compared to studies where high AMC (median 0.39–0.63) has correlated with poor prognosis [19, 22, 23, 24, 28, 40]. It is plausible that our patient cohort consisted mostly of the patients with poor prognosis if evaluated by AMC, and thus we could not find an AMC cutoff separating subgroups with different survival. In addition, ALC consists of all circulating lymphocytes rather than specific lymphocyte subtypes (B and T lymphocytes). Thus, the ratio of lymphocyte subtypes may be different in these high‐risk patients compared to heterogeneous patient groups, and therefore the ALC did not correlate with survival.

In our cohort, a cutoff level of 1.72 discriminated best the low and high LMR subgroups with different outcomes. The observation that low LMR is associated with inferior survival validates the previous findings [18, 19, 20, 21, 22, 23, 24, 25, 27, 29]. However, the cutoff level in our high‐risk cohort was lower than in previous studies being mostly around 2.6, suggesting that the cutoff may be dependent on the clinical risk group [18, 19, 20, 21, 22, 23, 24, 25, 26, 27, 29]. The treatment in our trial was also more intensive than the R‐CHOP regimen mostly used in the previous studies possibly affecting the outcome and thus interfering with the prognostic impact of LMR.

The reason for the association of low LMR with inferior survival is unclear. However, it is likely that low LMR is reflecting the imbalance between host inflammatory response and lymphoma microenvironment. Low ALC can be a sign of preexisting immunosuppression enabling lymphoma development, or a consequence of the immunological response to lymphoma. Macrophages in turn exhibit also protumoral functions, such as angiogenesis and extracellular matrix (ECM) remodeling in addition to antitumoral functions, and peripheral blood monocytes are regarded as an important reservoir of macrophage precursors, and a source of soluble mediators such as B lymphocyte stimulator (encoded by the *TNFSF13B* gene) to support the growth and survival of B cells [43, 44, 45, 46]. In contrast to the hypothesis that AMC is a surrogate biomarker for TAMs [47], but consistent with the findings by Matsuki et al. [26], we did not observe correlation between AMC and TAM content. One explanation could be that the lifespan of monocytes is usually only days compared to macrophages that live up to months [48]. Taken together, it is plausible to suggest that the relation of monocytes and lymphocytes (LMR) reflects the complex interplay between these cell types, and low LMR is a sign of their disadvantageous ratio.

## 5 CONCLUSION

In conclusion, our results in a uniformly treated patient cohort confirm the prior findings concerning the adverse prognostic impact of low LMR on survival in patients with aggressive B‐cell lymphoma. In addition, we show that low LMR translates to poor outcome also in young high‐risk patient population.

## CONFLICT OF INTEREST

Sirpa Leppä: Incyte*: consultancy and honoraria; Roche*: consultancy, honoraria, and research funding; Genmab*: research funding; Orion*: consultancy; Novartis*: consultancy, research funding, and honoraria; Bayer*: research funding; Celgene*: consultancy and research funding; Gilead*: consultancy. Harald Holte: Gilead*: consultancy and honoraria; Takeda*: consultancy and honoraria; Novartis*: consultancy; Genmab*: consultancy; Incyte*: consultancy and honoraria; Roche*: consultancy and honoraria. Other authors declare no conflict of interest. *Not related to this study.

## AUTHOR CONTRIBUTIONS

Heli Vajavaara designed and conceived the study, analyzed data, and wrote the manuscript. Suvi‐Katri Leivonen analyzed the data. Judit Jørgensen and Harald Holte provided samples. Sirpa Leppä designed and supervised the study and wrote the manuscript. All authors have read and approved the manuscript.

## ETHICS STATEMENT

Study was conducted according to the guidelines of the Declaration of Helsinki, and approved by the Ethics Committees in Finland, Norway, Denmark, and Sweden.
